# Implementation of the agmatine-controlled expression system for inducible gene expression in *Lactococcus lactis*

**DOI:** 10.1186/s12934-015-0399-x

**Published:** 2015-12-30

**Authors:** Daniel M. Linares, Patricia Alvarez-Sieiro, Beatriz del Rio, Victor Ladero, Begoña Redruello, Mª Cruz Martin, Maria Fernandez, Miguel A. Alvarez

**Affiliations:** Dairy Research Institute, Instituto de Productos Lácteos de Asturias (IPLA-CSIC), Paseo Rio Linares s/n, 33300 Villaviciosa, Spain

**Keywords:** *Lactococcus lactis*, Expression vector, Agmatine induction, *Myxococcus xanthus*, Prolyl-endopeptidase

## Abstract

**Background:**

*Lactococcus lactis* has been safely consumed in fermented foods for millennia. This Gram-positive bacterium has now become of industrial importance as an expression host for the overproduction of lipopolysaccharide-free recombinant proteins used as food ingredients, therapeutic proteins and biotechnological enzymes.

**Results:**

This paper reports an agmatine-controlled expression (ACE) system for *L. lactis,* comprising the lactococcal agmatine-sensor/transcriptional activator AguR and its target promoter P_*aguB*_. The usefulness and efficiency of this system was checked via the reporter gene *gfp* and by producing PEP (*Myxococcus xanthus* prolyl-endopeptidase), an enzyme of biomedical interest able to degrade the immunotoxic peptides produced during the gastrointestinal breakdown of gluten.

**Conclusion:**

The ACE system developed in this work was suitable for the efficient expression of the functional recombinant proteins GFP and PEP. The expression system was tightly regulated by the agmatine concentration and allowed high protein production without leakiness.

## Background

Heterologous protein production is a multi-billion dollar market of particular importance to manufacturers of biopharmaceuticals and enzymes for industrial use. Microbial production systems are often the best option for making such products given their ease of handling and high synthesis rates [1]. At present, *Escherichia coli* remains the first choice of host system given its high overexpression yields, ease of genetic handling, and the wealth of information available on this microorganism [2]. However, it is not without its drawbacks, such as the formation of inclusion bodies, the presence of an outer membrane that hampers secretion, its relatively complicated aerobic fermentation system, and the formation of endotoxins such as cell wall lipopolysaccharides [3]. The presence of bacterial endotoxins is one of the major concerns of regulatory agencies, and the need to add downstream steps to ensure their removal can make otherwise simple processes quite costly [4].

The Gram-positive bacterium *Lactococcus lactis* has emerged as an attractive alternative for the overproduction of recombinant proteins. Due to its long, safe history of use in dairy fermentations, this bacterium has been classified as a food grade microorganism ‘Generally Recognized As Safe’ (GRAS) by the US Food and Drug Administration (FDA), and has led it to receive ‘Qualified Presumption of Safety’ (QPS) status from the European Food Safety Authority (EFSA) [5]. In addition, it is an efficient secretor of extracellular recombinant proteins, has low protease activity (allowing for simplified purification processes), and a very simple metabolism that allows for rapid growth without aeration—all properties that facilitate scaling-up [4]. Moreover, *L. lactis* is likely to provide a good membrane environment for the overproduction of eukaryotic proteins [6]. Indeed, a number of eukaryotic membrane transporters, yeast mitochondrial proteins and human proteins have been heterologously expressed in this host [7]. Further, *L. lactis* is an efficient cellular factory able to turn out recombinant viral antigens, interleukins, allergens, virulence factors, bacteriocins and enzymes [8, 9]. It can even be genetically engineered to produce proteins from pathogenic species on its cell surface, and thus be used as a vector for the production and delivery of oral vaccines against HIV, cholera, malaria, human papillomavirus, stomach ulcers, tetanus and brucellosis [10–18].

For most of these applications, the availability of vectors that allow the cloning and expression of foreign genes is of paramount importance. Although the genetic accessibility and ease of handling of *L. lactis* lags far behind that of *E. coli*, the molecular biology techniques and genetic tools available for use with this bacterium have increased over recent years [9, 19]. So far, a number of inducible expression systems regulated by environmental factors have been documented, including the chloride-inducible expression cassette [20], the zinc-inducible expression system [21], the lactate-inducible P170 system [22], the heat shock-inducible system [23], systems based on sugar or peptide concentration-regulated promoters, and bacteriophage-derived promoters [24, 25]. However, some of these systems are less useful since they are controllable only to a limited extent, show low efficiency, or are associated with some degree of basal expression [21, 25, 26]. These limitations may be due to (1) the corresponding inducer being an essential nutrient or metabolite, the concentration of which in the culture cannot be fully controlled, (2) by being strongly sensitive to catabolite repression (i.e., certain sugar-inducible systems) [21, 25, 26], or (3) the promoter showing leaky activity. To date, the most widely used and potent gene expression system in *L. lactis* has been the nisin-inducible controlled expression (NICE) system. When added to the medium as an inducer, nisin binds to the membrane receptor NisK, which subsequently activates NisR by phosphorylation, and the activated NisR induces the *nisin A* promoter [27–30].

**Fig. 1:**
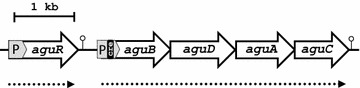
Genetic organization of the AGDI cluster of *L. lactis* CECT 8666. Physical map showing the cluster to be composed of five genes: *aguR* which encodes a transcription regulator, followed by *aguB*, *aguD*, *aguA* and *aguC,* which encode the proteins involved in the putrescine biosynthesis pathway (GenBank: AZSI00000000.1). The P_*aguR*_ and P_*aguB*_ promoters are shaded in *grey*, and the terminators indicated. The predicted transcripts are indicated below (*dotted arrows*). The *cre* site is shaded in *black*

It has been shown that the agmatine deiminase (AGDI) cluster of *L. lactis* subsp. *cremoris* CECT 8666 (formerly GE2-14) encodes the enzymatic activities responsible for the catabolism of agmatine to putrescine [31]. Briefly, *aguD* codes for the agmatine/putrescine antiporter, *aguA* encodes agmatine deiminase, *aguB* encodes putrescine transcarbamylase, and *aguC* encodes a specific carbamate kinase [32, 33]. Transcriptional analyses of these genes has shown the expression of *aguB*, *aguD*, *aguA* and *aguC* to be driven by the *aguB* promoter, and has confirmed the four genes to be co-transcribed as a single polycistronic mRNA [32, 33]. A *cre* site exists in the promoter of *aguB* which is transcriptionally regulated by carbon catabolite repression (CCR) mediated by the catabolite control protein CcpA [32, 33]. Also included in the AGDI cluster, upstream of the *aguBDAC* genes, is the *aguR* gene, which is transcribed constitutively under its own promoter (P_*aguR*_) (Fig. 1). We previously showed that *aguR* is a regulatory gene encoding a transmembrane protein (AguR) that acts as a one-component signal transduction system able to sense the extracellular agmatine concentration and trigger transcriptional activation of the *aguB* promoter (which drives expression of the *aguBDAC* operon) [34].

The present work reports the adaptation of the ACE system—an inducible gene expression system that involves *aguR* and the P_*aguB*_ promoter (the latter with its natural ribosome binding site)—to *L. lactis*. This was successfully tested via the production of green fluorescent protein (GFP) and *Myxococcus xanthus* prolyl-endopeptidase (PEP) [35, 36].

## Results

### Site-directed mutagenesis of the *cre* site of P_*aguB*_

**Fig. 2:**
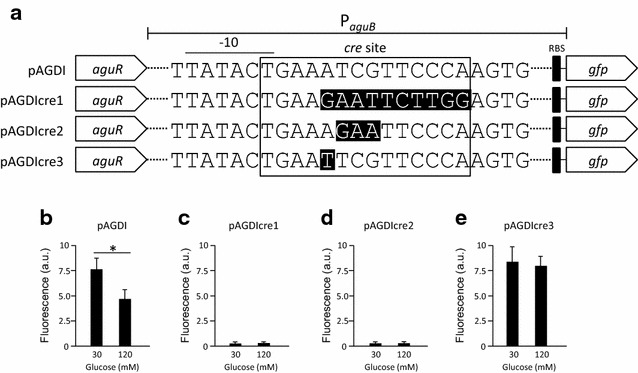
Generation of mutations in the *cre* site of P_*aguB*_ in the AGDI cluster of *L. lactis* CECT 8666, and their effect on promoter activity. **a** Genetic detail of the different *gfp* fusions made with the wild type P_*aguB*_ region (fusion pAGDI) and the derived promoters carrying different mutations in the *cre* site (fusions pAGDIcre1, pAGDIcre2 and pAGDIcre3). The introduced mutations are highlighted in *black*. *Dashed lines* indicate sequence discontinuities. **b**–**e** Promoter activity reported by GFP fluorescence (arbitrary units) for the wild type P_*aguB*_ and mutants assayed in the presence of 30 and 120 mM glucose under induction by 20 mM agmatine. Data represent the average of three independent experiments. *Bars* indicate standard deviations (*p < 0.05)

In previous work [34] we showed that P_*aguB*_ of *L. lactis* CECT 8666 drives expression of the *aguBDAC* operon in response to agmatine supplementation. Moreover, *aguBDAC* expression is regulated by CCR, mediated by the catabolite control protein A (CcpA) [33]. To develop an efficient gene expression system, CCR had to be inactivated so that glucose could be used as a carbon source. This allowed high bacterial cell densities to be obtained, and in turn high recombinant protein yields. For this we introduced arbitrarily directed mutations into the *cre* site (sequence 5′-TGAAATCGTTCCCA-3′; the nucleotides matching the *cre* consensus sequence are underlined) within P_*aguB*_ (see “Methods”). The pAGDI plasmid [33], containing the P_*aguR*_-*aguR*-P_*aguB*_ cassette fused to the reporter gene *gfp* (encoding green fluorescent protein [GFP]), was used to construct new plasmids to assess the effect of *cre* mutation on P_*aguB*_ activity. Using this plasmid as a template, and the primers indicated in Table 1, three new plasmids were generated which contained specific mutations in the *cre* site: pAGDIcre1 (containing 10 nucleotide mutations from positions 5–14 of the *cre* site), pAGDIcre2 (containing 3 nucleotide mutations from positions 6–8 of the *cre* site) and pAGDIcre3 (containing 1 nucleotide mutation at position 5) (Fig. 2a). The 10 and 3 nucleotide mutations (plasmids pAGDIcre1 and pAGDIcre2 respectively) both had a dramatic effect on P_*aguB*_: no activity was detected with either construct at any glucose concentration (Fig. 2c, d; compare with Fig. 2b [wild type]). The introduction of a single mutation (A > T) in the *cre* site (pAGDIcre3 construct) gave the expected result, i.e., expression was not repressed at 120 mM glucose (Fig. 2e). This mutated promoter was therefore selected for the construction of the expression vector.

**Table 1:** Oligonucleotides used in this study

Primer	Function	Sequence (5′ to 3′)
*mutf*	Mutation of pAGDIcre1 (F)	CACACACACGAATTCTTGGAGTGGGAAGTCAAATAACTATTT
*mutr*	Mutation of pAGDIcre1 (R)	CACACACACGAATTCTTCAGTATAACAAGGTTGATTTCT
*mutf1*	Mutation of pAGDIcre2 (F)	CACACACACGAATTCCCAAGTGGGAAGTCAAATAACTATTT
*mutr1*	Mutation of pAGDIcre2 (R)	CACACACACGAATTCTTTCAGTATAACAAGGTTGATTTCTT
*mutf4*	Mutation of pAGDIcre3 (F)	CACACACACGAATTCGTTCCCAAGTGGGAAGTCAAATAAC
*mutr4*	Mutation pAGDIcre3 (R)	CACACACACGAATTCAGTATAACAAGGTTGATTTCTTAAAAC
*prolF*	Cloning of *pep* (F)	CACACACACCCATGGCTTATCCAGCTACACGTGC
*prolR*	Cloning of *pep* (R)	CACACACACTCTAGATTAACGTCCTTGTGCAGC
*AgurBamHI*	Cloning P_*aguR*_-AguR-P_*aguB*_ cassette (F)	CCCCCCGGATCCGACAAGTTTGGCTCAGATTGCTTG
*PtcNco*	Cloning P_*aguR*_-AguR-P_*aguB*_ cassette (R)	CCCCCCATGGTGTTTATTCCTCCTGAATAAAATAG
*Expvfor1*	Insertion of His-Tag (F)	CACACACACCCATGGCTAATCGACTGCAGGAAAATTTATACTTCCAAGGTC
*Expvrev1*	Insertion of His-Tag (R)	CTATCAATCAAAGCAACACGTG
*GfF1*	Cloning of *gfp* (F)	CACACACACCCATGGAATTCAGTAAGGGAGAAGAACTTTTC
*GfR1*	Cloning of *gfp* (R)	CACACACACCTGCAGACTAGTTTTGTAGAGCTCATCCATGC

### Agmatine-induced heterologous expression of *gfp*

**Fig. 3:**
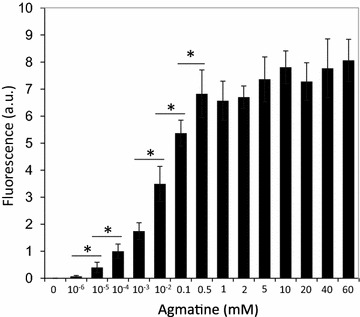
Sensitivity of the ACE system to agmatine. Strength of induction of *gfp* in *L. lactis* NZ9000 pACE-GFP. GFP protein activity was determined under a range of agmatine concentrations. Data represent the average of three independent experiments.* Bars* indicate standard deviations (*p < 0.05)

To verify the usefulness of the ACE system in the expression of heterologous genes, the reporter gene *gfp* was cloned into the pACE vector under the control of P_*aguB*_, thus generating the plasmid pACE-*gfp*. *L. lactis* NZ9000—a strain without the AGDI cluster—was transformed with pACE-*gfp*, thus resulting in *L. lactis* pACE-*gfp*. The presence of 10 mM agmatine in a culture of *L. lactis* pACE-*gfp* induced the expression of *gfp*, which was measured in terms of the fluorescence produced (7.87 arbitrary units) (Fig. 3). It should be noted that the expression of *gfp* was undetectable (<0.5 arbitrary units) in agmatine-uninduced cultures of *L. lactis* pACE-*gfp*. Fluorescence was also undetectable (<0.5 arbitrary units) in parallel agmatine-induced cultures of *L. lactis* NZ9000 harbouring the pACE vector (*L. lactis* pACE).

### Sensitivity of the ACE system to the inducer: dose–response curve

The production of GFP in cultures of *L. lactis* pACE-*gfp* induced using a range of agmatine concentrations (between 0 and 60 mM) was analysed by whole-cell fluorescence. The ACE expression system showed great sensitivity to very low agmatine levels; a significant increase in fluorescence was seen after induction with agmatine at concentrations as low as 10^−5^ mM (Fig. 3). Above this concentration, the induction levels increased in line with the agmatine concentration until a maximum induction level (fluorescence ~8 arbitrary units) was reached at 0.5 mM agmatine (no significant increases in induction were seen with concentrations of >0.5 mM). The absence of any leaky activity of the promoter P_*aguB*_, as verified by the absence of fluorescence in uninduced cultures (0 mM agmatine, Fig. 3), is remarkable.

### Heterologous production of GFP using the ACE system

The efficiency of the ACE system in overexpressing recombinant protein was tested with the reporter protein GFP. The expressed His-tagged GFP protein was purified using immobilized metal affinity chromatography (IMAC). The eluted protein fractions were examined by SDS-PAGE (data not shown) and their GFP activity. Pure protein with GFP activity was obtained in fraction two (of the four obtained); the yield was 47 % (Table 2). Fluorescence was found in the soluble fraction only (data not shown).

**Table 2:** Purification of GFP protein using the ACE system

Step	Total protein (µg)	Protein (µg ml^−1^)	Total activity (U mg^−1^)	Protein yield (%)^a^
Lysate	63,927	15,981	80	
Flow through	53,463	13,365	0	
Wash 1	3244	811	0	
Wash 2	4220	1055	0	
Elution 1	3049	6098	0	
Elution 2	235	471	171	47
Elution 3	55	111	0	
Elution 4	488	977	0	

^a^Recovery of fluorescence activity relative to the total activity of the soluble extract

### Heterologous overproduction of *M. xanthus* prolyl-endopeptidase and comparison with the NICE system

**Fig. 4:**
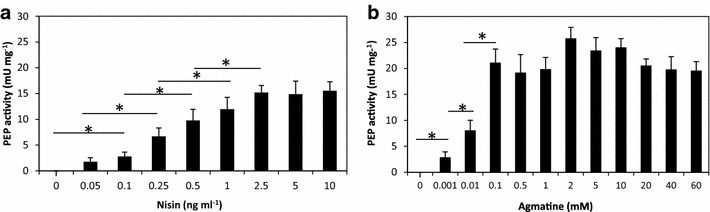
Comparison of PEP activity. **a** In the NICE system, PEP activity was monitored in *L. lactis* NZ9000 pNZ8048-*pep* cells induced at different nisin concentrations for 3 h after reaching OD_600_, while **b** in the ACE system, PEP activity was monitored in *L. lactis* NZ9000 pACE-*pep* cells induced with different agmatine concentrations (added to the culture medium before inoculation) after 7 h of growth. Data represent the mean of three independent experiments. *Bars* indicate standard deviations (*p < 0.05)

To confirm the usefulness and efficiency of the ACE system, the *pep* gene of *M. xanthus*, which encodes a prolyl-endopeptidase of biomedical interest, was cloned into appropriate plasmids for introduction into *L. lactis* NZ9000. The resulting *L. lactis* NZ9000 pACE-*pep* was induced with different agmatine concentrations (0, 0.001, 0.01, 0.1, 0.5, 1, 2, 5, 10, 20, 40 and 60 mM) and the PEP activity assayed. No activity was detected in cultures without agmatine, but was observed even with the lowest agmatine concentration tested (0.001 mM). Above this concentration, the induction level increased with the agmatine concentration until 0.1 mM agmatine (21.04 mU mg^−1^) (no significant increase in PEP activity was obtained with concentrations of >0.1 mM) (Fig. 4). PEP activity was sought in the soluble and insoluble fractions, but was only seen in the former.

For comparison, PEP was also produced using the NICE system at different nisin concentrations (0, 0.05, 0.1, 0.25, 0.5, 1, 2.5, 5, 10 ng ml^−1^) (Fig. 4). The activity increased with the nisin concentration until 2.5 ng ml^−1^. The highest specific activity obtained (15.2 mU mg^−1^) was lower than that obtained with the ACE system (21.04 mU mg^−1^). Again, all PEP activity was observed in the soluble fraction.

### Influence of agmatine on *Lactococcus lactis* growth

Since high induction concentrations of agmatine were tested in the present work, assays were performed to see whether these affected bacterial fitness. *L. lactis* cultures were grown in liquid GM17 supplemented with 0, 0.001, 0.01, 0.1, 0.5, 1, 2, 5, 10, 20, 40 or 60 mM agmatine. Similar growth curves were obtained (data not shown). Table 3 shows the OD_600_, μ_max_ and pH values and lactate concentrations reached after 24 h of incubation. Growth slightly decreased with increasing concentration of agmatine. Although the 0.5 and 0.1 mM agmatine concentrations led to the highest GFP and PEP activities, no differences in OD_600_ were observed compared to the uninduced cultures. Organic acids and sugars were analyzed by HPLC, and no differences observed in the presence or absence of agmatine (data not show). Agmatine had a weak effect on *L. lactis* cell viability after 24 h of exposure; optical density values suitable for protein production were obtained even at high agmatine concentrations.

**Table 3:** Effect of agmatine on growth, μ_max_, pH and production of lactic acid

Agmatine (mM)	OD_600_	μ_max_	pH	Lactic acid (mM)
0	5.12	0.70	4.67	80.21
0.0001	4.86	0.69	4.69	81.15
0.001	4.44	0.67	4.7	80.10
0.01	3.9	0.62	4.72	80.35
0.1	4.5	0.65	4.77	79.83
0.5	4.11	0.65	4.79	79.07
1	4.11	0.66	4.79	78.55
2	4.11	0.63	4.79	78.22
5	4.31	0.60	4.77	78.62
10	3.89	0.61	4.77	78.89
20	4.12	0.61	4.78	76.14
40	3.65	0.58	4.8	73.49
60	3.85	0.59	4.78	67.40

## Discussion

*Lactococcus lactis* has long been used in the food industry and has emerged as a cost-effective cellular factory for the production of proteins of interest [4]. At present, the genome sequences of several strains have been elucidated, and the genetic tools available for use with lactic acid bacteria (LAB) are ever increasing the number of fully lipopolysaccharide-free recombinant proteins that can be produced [3, 37].

The control of gene expression is critical in achieving high protein yields. For example, it is essential for ensuring the conservation of energy for the production of biomass prior to the directed overproduction of the target protein, and in controlling the production of products that might be toxic to the host cell (i.e., membrane proteins, autolysins, lysis-related proteins from phages) [3, 38]. Systems that allow for well controlled inducible expression, and that allow no basal expression, are essential in setting production rates. A number of inducible expression systems for *L. lactis* are available [20–22, 25, 39], however, the use of some of them, especially in large-scale fermentations, may be hampered by the low-level induction achieved, high background expression before induction (leaky activity), and/or a lack of control over the inducer [21, 25, 26, 39]. Such drawbacks are not suffered with the NICE system, probably the most commonly used regulated expression system for Gram-positive bacteria [27–29, 40]. However, when using a host strain other than *L. lactis* NZ9000, the NICE system requires the regulatory genes *nisR* and *nisK* to be supplied *in trans*. Thus, although the NICE system has been optimised to be used as a single-plasmid expression vector [41], its exploitation nearly always requires the integration of *nisR* and *nisK*, either in the chromosome of the host strain or via their cloning into a separate plasmid (the dual-plasmid strategy).

The present work describes an alternative system based on the *aguR*/P_*aguB*_ cassette (the regulatory part of the AGDI cluster of *L. lactis* CECT 8666) for agmatine-controlled gene expression in *L. lactis* using a vector that includes all the elements required. It should be noted that this system requires the expression of no additional genes supplied *in trans*. The developed vector relies on the regulatory transmembrane protein AguR, which responds to extracellular agmatine, and in so doing triggers the induction of gene expression via P_*aguB*_ [34]. It was previously shown that P_*aguB*_ of *L. lactis* CECT 8666 contains a *cre* site involved in CCR and that this is repressed by glucose concentrations of >30 mM [33]. Since higher concentrations of glucose may be required in culture media to obtain the densities of bacterial cells needed to provide high recombinant protein yields, this repression mechanism was eliminated in the present work through the introduction of a single A > T mutation at position 5 in the *cre* site. Larger mutations of either 3 or 10 nucleotides completely impaired the activity of the promoter, most likely by preventing some additional regulatory signal. Other authors have also shown CCR to be relieved when single mutations occur in the *cre* site of CCR-controlled genes in *L. lactis*. For example, a single mutation in the *cre* site of the *celB* promoter allows fully active transcription of the cryptic *cel* cluster involved in lactose utilization in *L. lactis* MG1363 [42]. Similarly, two single mutations in the *cre* site of the *ptcC* promoter do away with the glucose-repressor effect and allow cells to constitutively metabolize cellobiose [43].

An expression vector combining the one-component signal transduction system, i.e., *aguR* and the *aguB* promoter, followed by convenient cloning sites for introducing the gene of interest, was constructed. An important feature of the developed pACE vector is the possibility of fusing a His-tag to the protein of interest by cloning the encoding gene in frame into the *Nco*I-*Pst*I sites. His-tags have been used in other *L. lactis* expression vectors previously shown to perform efficiently in the immunodetection and purification of proteins [6, 44]. We here confirm the functionality of the His-tag in purifying the GFP (protein yield 47 %).

The control of the ACE system was assessed via the expression of *gfp*, the reporter gene coding for GFP. Strong fluorescence was seen in the presence of ≥0.5 mM agmatine (8 arbitrary units compared to 0 in uninduced cells). Interestingly, the system was associated with no basal expression, indicating P_*aguB*_ to have no leaky activity. Further, agmatine is not present in common culture media, thus allowing for tight control over the gene to be expressed. Neither is it found in milk nor any derivative dairy environment where *L. lactis* occurs [45]. The optimal moment of induction, which can change from one overexpressed gene to another, needs to be evaluated. In the ACE system, the inducer agmatine can be added when the culture medium is prepared. The AguR/P_*aguB*_ cassette, on which this expression system is based, is the regulatory part of the AGDI cluster of *L. lactis* [33] and is not active until the transition between the exponential and stationary phases is reached (5–6 h of culture) [46]. Thus, even when agmatine is supplied to the culture medium, the time of net expression would lie between 5 and 7 h after culturing began. This induction time is comparable to that associated with the NICE system (2–3 h after adding nisin). The addition of the inducer at the beginning of culturing avoids the problem of monitoring the culture’s optical density to determine the optimal moment for induction. Moreover, the abolition of sampling and inducer addition steps may prevent contamination, which could have serious economic consequences in industrial protein production. As seen for nisin, agmatine affected cell viability and caused a 20 % reduction in bacterial yield. However, the final OD (>3.3) of agmatine-induced cultures was still optimal for industrial protein production.

The price of the inducer is important in large scale fermentations. That of agmatine varies widely, depending on the supplier, but even the cheapest found (marketed as dietary supplement) worked properly as an inducer (data not shown). Certainly, it was much cheaper than nisin.

An agmatine induction system combining AguR and P_*aguB*_ of *Enterococcus faecalis* was earlier used to develop an expression vector suitable for the latter species [47]. However, the proposed lactococcal ACE system shows some differences to the *E. faecalis* system: (1) the *aguR* gene is in the same orientation as P_*aguB*_ (reflecting the corresponding organization of the AGDI cluster in each system), (2) the highest expression rate is reached at ≥0.5 mM agmatine in the ACE system but at ≥60 mM in the *E. faecalis* system, and (3) the level of induction (as determined by fluorescence) is less than that achieved with the *E. faecalis* system.

In the present work, the performance of the ACE system for the heterologous production of the *M. xanthus* PEP was compared to that obtained using the NICE system, the most widely used and potent gene expression system in *L. lactis* [40]. The proposed system achieved higher PEP activity (*circa* 38 %) under similar laboratory conditions. The observed differences might be related to the characteristics of each promoter, or to the effect on signal transduction of the two-component NICE system compared to the one-component ACE system. More studies are required to understand how the agmatine signal is transduced.

In summary, the present results confirm the ACE system as an attractive candidate for high level recombinant protein production. The lactococcal *aguR*/P_*aguB*_ system can effectively control the expression of genes in response to agmatine in *L. lactis* without any basal expression, and combines both the expression cassette and regulatory gene in one plasmid. This vector expands the genetic toolbox available for this species, and could be a powerful and straightforward alternative system for overexpressing proteins in lactococcal strains lacking *nisR* and *nisK*. It might also be used to complement the NICE system and be used in co-expression.

## Conclusions

The present work describes the construction of a *L. lactis* agmatine-controlled expression system based on the *aguR*/P_*aguB*_ cassette of the putrescine biosynthesis gene cluster. A single mutation of the *cre* site in P_*aguB*_ abolished the CCR of this promoter. This system was assessed by expressing the reporter gene *gfp*, and fluorescence was found strictly dependent on the agmatine concentration added to the culture medium, with maximum induction occurring at 0.5 mM agmatine (7 arbitrary units compared to 0 in uninduced cells). An important potential benefit of this system is the lack of leaky activity associated with it, and the fact that gene expression can be tightly controlled via the addition of the appropriate concentration of agmatine. The pACE vector allowed the agmatine-inducible expression of the gene encoding *M. xanthus* PEP, an enzyme that can degrade the immunotoxic peptides of gluten breakdown. Moreover, enzymatic activity was greater than that obtained with the NICE expression system. The addition of a His-tag to the pACE vector renders the system suitable for protein purification and immunodetection purposes. Together, these findings suggest that the ACE expression system could be a very valuable addition to the *L. lactis* genetic toolbox, and offers a straightforward, alternative inducible gene expression system that to be used in functional studies and in the large-scale production of recombinant proteins.

## Methods

### Bacterial strains and growth conditions

*L. lactis* CECT 8666 (formerly GE2-14) and *L. lactis* NZ9000 were grown at 30 °C in M17 medium (Oxoid, Basingstoke, United Kingdom) supplemented with 30 mM glucose (GM17). When required, agmatine (Sigma-Aldrich, St. Louis, MO, USA) was added to the medium. Chloramphenicol (5 μg ml^−1^) was added as required for plasmid maintenance. For overexpression using the NICE system, cultures of *L. lactis* NZ9000 in exponential phase (OD_600_ = 0.4–0.5), grown in GM17, were induced for 3 h with various nisin concentrations (0, 0.05, 0.1, 0.25, 0.5, 1, 2.5, 5 and 10 ng ml^−1^) (Sigma-Aldrich). Solid media were prepared by adding 2 % (w/v) agar (Merck, Darmstad, Germany). Microbial growth was examined in all cultures by measuring absorbance at 600 nm (OD_600_) using a spectrophotometer (Eppendorf, NY, USA). The pH of the samples was measured using a CRISON miCropH 2001 pH meter (Crison Instruments S.A., Barcelona, Spain). The maximum specific growth rate (μ_max_) was determined experimentally in the exponential growth phase, as described by O´Sullivan and Condon [48].

### DNA manipulation

The procedures used for DNA manipulation and recombination were essentially those described by Sambrook et al. [49]. Table 1 lists the sequences of primers used. Genetic constructs were achieved in *L. lactis* NZ9000. The isolation of *L. lactis* plasmids and total DNA, and the transformation procedures followed, were as previously described [50]. All plasmid constructs were verified by nucleotide sequencing at Macrogen Inc. (Seoul, Republic of Korea). All enzymes for DNA technology were used according to the manufacturer’s specifications.

### Construction of plasmids

**Fig. 5:**
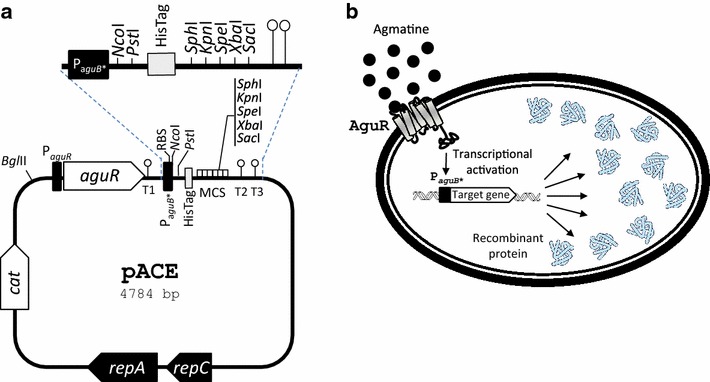
**a** Genetic map of the pACE expression vector. *repC* and *repA*, replication genes; *cat*, chloramphenicol resistance gene; *aguR*, gene encoding the regulatory agmatine-sensor-regulator protein AguR; P_*aguR*_, *aguR* promoter; RBS, ribosome binding site; T1, T2 and T3, transcription terminators (ΔG = −10.3, −9.7 and −8.3 kcal/mol respectively); P_aguB*_, *aguB* agmatine-inducible promoter carrying the A > T mutation in the fifth nucleotide of the *cre* site; MCS, multicloning site; HisTag; C-terminal histidine tag. Representative restriction sites are indicated. **b** Overview of the AguR/P*aguB* expression system including its components and role. AguR is the transmembrane agmatine (*black filled circles*) sensor and response regulator that activates P_*aguB*_, thereby triggering the massive overproduction of the recombinant target protein

The core of the lactococcal vector pNZ8048 [29]—which includes the replication cassette and the chloramphenicol resistance marker—was used as a starting point for the construction of the pACE vector. First, a fragment of the AGDI cluster from *L. lactis* CECT 8666 (Table 4), including the *aguR* promoter (P_*aguR*_), the *aguR* gene, and the *aguB* promoter (P_*aguB*_) carrying the mutation in the *cre* site, was PCR-amplified (using pAGDIcre3 as a template) and cloned into the *Bgl*II-*Nco*I sites of pNZ8048. Subsequently, a fragment including the multicloning site and a histidine tag encoding 10 consecutives histidines (His-tag) was amplified from plasmid pNZErmC [6] and cloned into the *Nco*I-*Xba*I sites of the previous construct, thus yielding vector pACE (Fig. 5). This vector offers the option to fuse in-frame the gene encoding the protein of interest to a C-terminal His-Tag by cloning the target gene into the *Nco*I-*Pst*I sites. Thus, this vector could be used for immunodetection or purification of the proteins encoded by overexpressed genes. The target gene could also be cloned without the His-tag for use in functional studies.

**Table 4:** Strains and plasmids

Strain/plasmid	Characteristics	References
Strains		
*L. lactis* CECT8666 (formerly GE2-24)	Isolated from an artisanal cheese, containing AGDI cluster	[31]
*L. lactis* NZ9000	Expression host for NICE system, lacking AGDI cluster	[29]
Plasmids		
pNZ8048	Lactococcal plasmid containing the *nisA* promoter and the Cm^r^	[29]
pNZErmC	pNZ8048 derivative containing the His-tag and the Cm^r^	[6]
pUC57-*pep*	pUC57 derivative containing the *pep* gene from *M. Xanthus* and the Ap^r^	[36]
pAGDI	pNZ8048 derivative bearing the P_*aguR*_-*aguR*-P_*aguB*_-*gfp* fusion and the Cm^r^	[34]
pAG2	pNZ8048 derivative bearing the P_*aguB*_-*gfp* fusion and the Cm^r^	[33]
pAGDIcre1	pAGDI derivative bearing the mutated P_*aguB*_ and the Cm^r^	This work
pAGDIcre2	pAGDI derivative bearing the mutated P_*aguB*_ and the Cm^r^	This work
pAGDIcre3	pAGDI derivative bearing the mutated P_*aguB*_ and the Cm^r^	This work
pACE	Vector for ACE system containing the P_*aguR*_-*aguR*-P_*aguB*_ cassette from pAGDIcre3 and the Cm^r^	This work
pACE-*gfp*	pACE derivative harbouring the *gfp* gene from pAG2 and the Cm^r^	This work
pNZ8048-*pep*	pNZ8048 derivative harbouring the *pep* gene from pUC57-PEPand the Cm^r^	This work
pACE-*pep*	pACE derivative harbouring the *pep* gene from pNZ8048-PEP and the Cm^r^	This work

P_*aguR*_, *aguR* promoter; P_*aguB*_, *aguB* promoter; *pep*, prolyl endopeptidase gene; Cm^r^, chloramphenicol resistance marker; Ap^r^, ampicillin resistance marker

For the heterologous expression of GFP, the *gfp* gene (amplified from pAG2 [34]) was inserted into the *Nco*I-*Pst*I sites of pACE, thus generating the plasmid pACE-*gfp.* To produce PEP using the ACE system, the *pep* gene was PCR-amplified from pNZ8048-*pep* and cloned into the *Nco*I-*Xba*I sites in the pACE vector, resulting in the pACE-*pep* plasmid. To produce PEP using the NICE system, the *pep* gene was released from plasmid pUC57-*pep* [36] as a *Nco*I-*Xba*I fragment and cloned into the same sites in pNZ8048 under the control of the *nisA* promoter [29], thus generating plasmid pNZ8048-*pep*.

### Directed mutagenesis of the *cre* site of P_*aguB*_

Modification of the *cre* site was achieved by in vitro site-directed mutagenesis. Mutation(s) were introduced by PCR using two divergent primers (Table 1) spanning the *cre* site of P_*aguB*_ and containing the desired mutation(s). Each primer was complementary to the opposite strand of the pAGDI vector, which was used as template to generate plasmids pAGDIcre1, pAGDIcre2 and pAGDIcre3 containing the specific mutations in the *cre* site. The pAGDI plasmid was first methylated with Dam methylase and S-adenosyl methionine following the manufacturer’s instructions (New England Biolabs, Hertfordshire, UK). Phusion High-Fidelity DNA Polymerase (New England Biolabs) was used to amplify both plasmid strands with high fidelity. The PCR thermoycling conditions were as follows: initial denaturation (98 °C for 30 s), 32 cycles of amplification (98 °C for 10 s; 55 °C for 30 s; and 72 °C for 2.5 min) plus a final extension step (72 °C for 7 min). An EcoRI target site was included in the primers so that the obtained amplicons could be digested with *Eco*RI and self-ligated. Before transformation in *L. lactis* NZ9000, the ligation mixture was treated with *Dpn*I order to digest the original, Dam methylated pAGDI plasmid used as a template.

### Measurement of green fluorescence

For whole-cell fluorescence measurements, overnight cultures of *L. lactis* NZ9000 harbouring either pACE or pACE-*gfp* were transferred (1 %) to fresh medium (GM17) supplemented with different agmatine concentrations (0–60 mM) and grown for 7 h. Equal amounts of cells were harvested, washed, and then resuspended in 50 mM potassium phosphate buffer, pH 7.2, as previously described [6]. GFP emission was measured in a volume of 200 μl of cells, using a Cary Eclipse fluorescence spectrophotometer (Varian Inc., Palo Alto, CA, USA) at an excitation wavelength of 485 nm and an emission wavelength 530 nm. To facilitate direct comparisons, the bacterial cultures used for GFP fluorescence measurements contained similar amounts of cells (estimation was made based on OD_600_). Background fluorescence levels were assessed using non-fluorescent control cells (lacking the *gfp* gene), and these values subtracted from the experimental results.

### Prolyl-endopeptidase assay

PEP activity was determined using a synthetic substrate, succinyl-Ala-Pro-*p*-NA (NA, nitroanilide) (Bachem, Bubendorf, Switzerland), as previously described [36] with slight modifications. Bacterial cultures (10 ml) of *L.lactis* pNZ8048-*pep* (induced with nisin for 3 h after the cells reached an OD_600_ of 0.6) and *L. lactis* pACE-*pep* (induced with 20 mM agmatine [added when the culture medium was prepared] and grown for 7 h) were harvested by centrifugation (8000*g* for 10 min), washed twice, and resuspended in 2 ml of 50 mM phosphate buffer, 0.2 M NaCl, pH 7.5. The samples were then disrupted using 200 mg glass beads (<106 µm) (Sigma-Aldrich) in a Fast-Prep FP120 Instrument (Thermo Savant-BIO101/Q-Biogen, CA, USA) for 6 × 30 s at power setting 4.5 (with intermittent cooling). Cell debris was removed by centrifugation (10,000*g* for 30 min at 4 °C) and the supernatant used in activity assays. The assay mixture contained 625 μl of 50 mM phosphate buffer (pH 7.5), 0.2 M NaCl, 125 μl substrate (1.2 mM), and 250 μl of cell extracts. The reaction was stopped by adding 500 μl of 20 % trichloroacetic acid. The samples were then centrifuged (10,000*g* for 10 min) and the release of the *p*-NA spectrophotometrically detected at 410 nm in a U-2800 Digilab Hitachi spectrophotometer (HitachiHigh-Technologies Corporation, Tokyo, Japan). One activity unit was defined as the amount of enzyme required to release 1 μmol of *p*-NA per min under the assay conditions. Assays were performed in triplicate. Specific enzyme activity was expressed as milliunits per milligram of protein. The protein concentration was measured using a Pierce BCA Assay Kit (Thermo Fisher scientific) following the manufacturer’s indications.

### Purification His-tagged protein

Purification of the His-tagged GFP protein was performed by immobilized metal ion affinity chromatography (IMAC). Cells (200 ml) induced with 10 mM agmatine were harvested by centrifugation at 8000*g*, at 4 °C for 10 min after 7 h of growth. The supernatant was discarded and cells washed twice and resuspended in 4 ml phosphate buffer (50 mM pH 7.5). They were then disrupted using a French Press operating at 2.3 kbar [Constant Cell Disruption Systems (Low March, Daventry, Norttants, UK)]. Cell debris was removed by centrifugation (10,000*g* for 30 min at 4 °C). Imidazole was then added to a concentration of 10 mM, and the resulting samples employed in protein purification using the QIAexpressionist kit (Quiagen, Madrid, Spain) following the manufacturer’s instructions. Aliquots of collected fractions were analyzed by SDS-PAGE using 12 % acrylamide gels to determine the purity of the His_6_-taggeted proteins. Their GFP activities and protein concentrations were determined using the protocols mentioned above to determine the protein yield.

### Soluble and insoluble protein fractions

Soluble and insoluble proteins fractions were prepared following the protocol described by Cano-Garrido et al. [51]. Samples (10 ml) of bacterial cultures grown at different agmatine concentrations were pelleted by centrifugation at 8000*g* at 4 °C for 10 min and the sediment resuspended in 1 ml of the appropriate buffer depending on the protein expressed (GFP—phosphate buffer 50 mM, pH 7.5; PEP—phosphate buffer 50 mM, pH 7.5, 0.2 M NaCl). The samples were then disrupted with 200 mg glass beads (<106 µm) (Sigma-Aldrich) in a Fast-Prep FP120 Instrument (Thermo Savant-BIO101/Q-Biogen, CA, USA) for 6 × 30 s at a power setting of 4.5 (with intermittent cooling). Total cell extracts were centrifuged at 15,000*g* at 4 °C for 15 min. Finally, the insoluble fractions were resuspended in 1 ml of the appropriate buffer and fluorescence and PEP activity monitored in both the soluble and insoluble-resuspended fractions.

### Determination of organic acids and sugars by HPLC

Sugar and organic acid concentrations were determined using a chromatographic system composed of an Alliance 2690 module injector, a Photodiode Array PDA 996 detector, and a 410 Differential Refractometer detector, all controlled with Millennium 32 software (Waters, Milford, MA, USA). Supernatants (50 μl) were isocratically separated in a 300 × 7.8 mm HPX-87H Aminex ion-exchange column (Hewlett Packard, Palo Alto, CA, USA) protected by a cation H+ Microguard cartridge (BioRad, Laboratories, Richmond, CA, USA), at a flow rate of 0.7 ml/min and a temperature of 65 °C. Sulphuric acid (3 mM) was used as the mobile phase. A PDA 996 detector at 210 nm was used to identify and quantify the organic acids detected, whereas the sugars were analyzed with a 410 Refractometer. Solutions of lactic and acetic acids, glucose, galactose, lactose, and sucrose were used as standards in the identification and quantification procedure.

### Statistical analysis

The Student *t* test was used to examine differences between groups. Significance was set at *p* < 0.05.

## Abbreviations

ACE: agmatine-controlled expression
AGDI: agmatine deiminase
CCR: carbon catabolite repression
GFP: green fluorescence protein
NICE: nisin-inducible controlled expression
PEP: prolyl-endopeptidase
